# Identification of sequence polymorphisms at 58 STRs and 94 iiSNPs in a Tibetan population using massively parallel sequencing

**DOI:** 10.1038/s41598-020-69137-1

**Published:** 2020-07-22

**Authors:** Dan Peng, Yinming Zhang, Han Ren, Haixia Li, Ran Li, Xuefeng Shen, Nana Wang, Erwen Huang, Riga Wu, Hongyu Sun

**Affiliations:** 1 Faculty of Forensic Medicine, Zhongshan School of Medicine, Sun Yat-Sen University, No. 74 Zhongshan Road II, Guangzhou, 510080 Guangdong People’s Republic of China; 2 Guangdong Province Translational Forensic Medicine Engineering Technology Research Center, Sun Yat-Sen University, Guangzhou, 510080 People’s Republic of China

**Keywords:** Genetics, Molecular biology

## Abstract

Massively parallel sequencing (MPS) has rapidly become a promising method for forensic DNA typing, due to its ability to detect a large number of markers and samples simultaneously in a single reaction, and sequence information can be obtained directly. In the present study, two kinds of forensic genetic markers, short tandem repeat (STR) and identity-informative single nucleotide polymorphism (iiSNP) were analyzed simultaneously using ForenSeq DNA Signature Prep Kit, a commercially available kit on MPS platform. A total of 152 DNA markers, including 27 autosomal STR (A-STR) loci, 24 Y chromosomal STR (Y-STR) loci, 7 X chromosomal STR (X-STR) loci and 94 iiSNP loci were genotyped for 107 Tibetan individuals (53 males and 54 females). Compared with length-based STR typing methods, 112 more A-STR alleles, 41 more Y-STR alleles, and 24 more X-STR alleles were observed at 17 A-STRs, 9 Y-STRs, and 5 X-STRs using sequence-based approaches. Thirty-nine novel sequence variations were observed at 20 STR loci. When the flanking regions were also analyzed in addition to target SNPs at the 94 iiSNPs, 38 more alleles were identified. Our study provided an adequate genotype and frequencies data of the two types of genetic markers for forensic practice. Moreover, we also proved that this panel is highly polymorphic and informative in Tibetan population, and should be efficient in forensic kinship testing and personal identification cases.

## Introduction

Several genetic markers have been introduced to forensic genetics to clarify the problems of kinship analysis and personal identification. Short tandem repeats (STRs) and single nucleotide polymorphisms (SNPs) are commonly used genetic markers in present forensic cases^1,2^.

STRs, usually 2–6 bp in length, are commonly typed with the amplified fragment length polymorphism (Amp-FLP) strategy combining fluorescently labelled multiplex PCR and capillary electrophoresis (CE)^3^. Allele calling can thus be inferred from fragment length by comparison with a locus specific allelic ladder that has been previously sequenced, where the number of repeat units is distinct^2^. Thus, each allele is regarded as a length-based (LB) allele using this approach. With the advancement of sequencing technologies over the last decade, the existence of sequence structure variations in alleles with the same length has been uncovered^4^.

SNPs, which could be amplified with smaller amplicons, are bi-allelic genetic markers with lower mutation rates compared with STRs^5^. Several autosomal SNP marker sets and detection methods, such as single-base extension, chip-based microarrays, and allele-specific hybridization arrays, have been developed to compensate for the relatively weaker discrimination power of single loci caused by the bi-allelic nature of the human genome^5–7^. However, these methods are not widely used in forensic practice due to the requirement of higher DNA inputs or the limited ability to detect a vast number of SNP loci in a single reaction^8^.

Different from detection methods mentioned above, massively parallel sequencing (MPS), also known as next-generation sequencing (NGS), provides new technology for forensic genetic marker typing. Numerous markers and samples can be investigated simultaneously with MPS, and there is no need to consider the problem of the size overlapping of amplified fragments or the availability of fluorescent labels as CE method does. For STRs, both length and sequence data can be achieved; thus, allele calling may be more informative and the allele’s sequence characteristics are identified, resulting in sequence-based (SB) alleles. For SNPs, not only the target SNPs but also the variations in the flanking regions can be identified simultaneously, and form the potential microhaplotype^9,10^. Thus, more alleles can be identified based on the analysis of full sequences of SNPs.

This new technology puts forward new challenges to researchers. First of all, the immense variable and complex data produced by MPS platforms is hard to be analysed manually. Meanwhile, the software packages developed for LB datasets are not efficient anymore. New bioinformatic methods are required to process and interpret these extensive data. An optimal package for MPS data analysis needs to be accurate, time-saving and easy to operate. Several packages has been published to make this process convenient for forensic uses, such as TSSV^11^, STRait Razor^12,13^, STRinNGS^14^, SEQ Mapper^15^, FDStools^16^ et al. Sequencer manufacturers also carried out supplementary analysis packages to fit for the data produced by their sequencers, such as ForenSeq Universal Analysis Software^17^ (UAS, Illumina, San Diego, CA) and Ion Torrent Suite Software Plugins^18^ (Thermo Fisher Scientific, South San Francisco, CA).

Moreover, the LB nomenclature of CE method for STR is not suitable for the complex sequence variations detected by MPS platforms. It is urgent to know how the MPS data should be analysed and reported, what connections do these data have with LB alleles, and how to record and search such datasets in a database^4^. Some researchers have tried to answer these questions^19,20^ but a perfect nomenclature is still under development. A unified minimal nomenclature of the complex sequences obtained by MPS technologies was recommended by the International Society for Forensic Genetics (ISFG) in 2016^21,22^ to facilitate communication between laboratories and to make this data backward compatible with LB data produced on CE platform. In early 2019, the STRAND Working Group was formalized to discuss the expanding and advancing topics of STR sequence nomenclature^23^. Quality control of string sequences and alleles has also been suggested by ISFG^24^.

Aiming to facilitate MPS in forensic genetics practice, several commercial and custom STR typing systems have been developed based on different MPS platforms for different purposes^25–28^. The ForenSeq DNA Signature Prep Kit (Illumina, San Diego, CA) is one of the library preparation kits that simultaneously targets the sequences of Amelogenin, 27 autosomal STRs (A-STRs), 24 Y-STRs, 7 X-STRs, and 94 identity informative SNPs (iiSNPs) in Primer Mix A (DPMA), with the option to include an additional 56 ancestry informative SNPs (aiSNPs) and 22 phenotype informative SNPs (piSNPs) in Primer Mix B (DPMB). A pair-ended sequencing will be performed after the library preparation and then the raw data will be imported to UAS to analyse automatically. Validations using the ForenSeq Signature system have demonstrated its advantages in forensic practice relative to other library preparation kits^29–33^, but the knowledge of alleles and genotype frequencies of these 58 STRs is still inadequate for accurate lineage analysis^34–39^ and is not sufficient for population genetic studies, which limits its utility in forensic casework.

The Tibetan ethnic group is one of the oldest ethnic groups in China and in South Asia, and the culture of ancient Tibet was thrived from the tenth to the sixteenth century. The Tibetan ethnic group includes a population of 6.3 million people according to the 2010 Chinese census and they have resided mainly on the highest plateau in the world, the Qinghai-Tibetan Plateau (average elevation ranges from 4,000 to 5,000 m), for hundreds of generations. They have a distinctive language, clothing, customs, religious characteristics from other Chinese or South Asian ethnic groups^40^ and can be further classified as Wei Tibetan, Kangba Tibetan and Amdo Tibetan^41^ by linguistics. The diversities of LB STR alleles using CE methods have been reported in some studies^42,43^, but the polymorphisms of SB STR and SNP alleles on MPS platforms using the Forenseq system have not been researched in this ethnic group.

In this study, a Tibetan population from Lhasa (Wei Tibetan), the capital of the Tibetan Autonomous Region, was analysed. Sequence variations in 58 STRs and 94 iiSNPs, and population data from these two kinds of markers were reported. Parameters of evidence weight were also performed.

## Results

### Sequencing quality and concordance analysis

**Figure 1:**
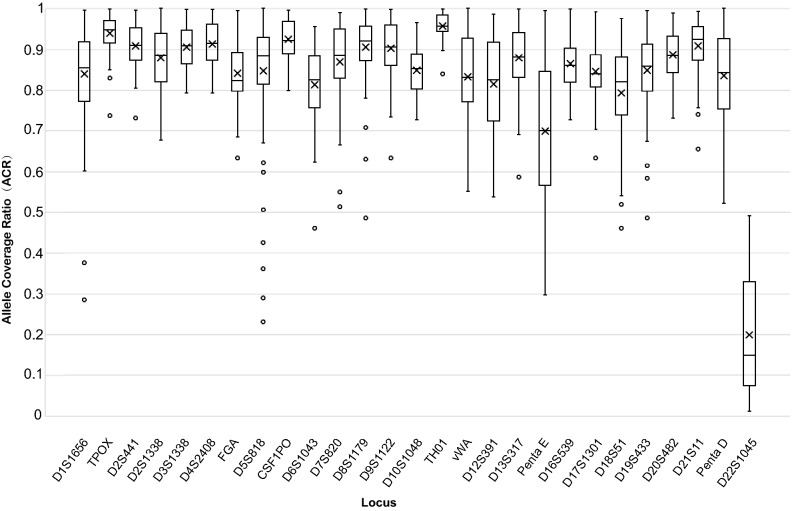
Allele coverage ratios of 27 A-STRs.

The average cluster density was 740 k/mm^2^, while the average total number of reads was 96,239 and 110,292 for each sample for the two runs (Supplementary Table S1). Concordance analysis of two software packages (UAS and STRait Razor v2s) showed the same allele calling based on length. The LB alleles were in concordance with corresponding CE results for the 23 shared STR loci from the Forenseq system and Goldeneye DNA ID System 25A amplification system (Peoplespot SciTech Incorporation, Beijing, China) except D22S1045. Allele imbalance was observed at D22S1045 and all of the ACRs from D22S1045 were lower than 0.50 (range from 0.0113 to 0.4918) (Fig. 1), which led to some miscalling of heterozygotes as homozygotes. Considering that allele genotypes of the D22S1045 locus were questionable, D22S1045 were discarded for the following statistics. Sequence-based average ACRs of the other 26 A-STRs ranged from 0.6996 (Penta E) to 0.9572 (TH01) (Fig. 1).

A-STRs dataset were verified and accepted by STRidER (https://strider.online/) with the assigned accession number STR000149^22^.

### Novel alleles and STR allele sequence variations

**Figure 2:**
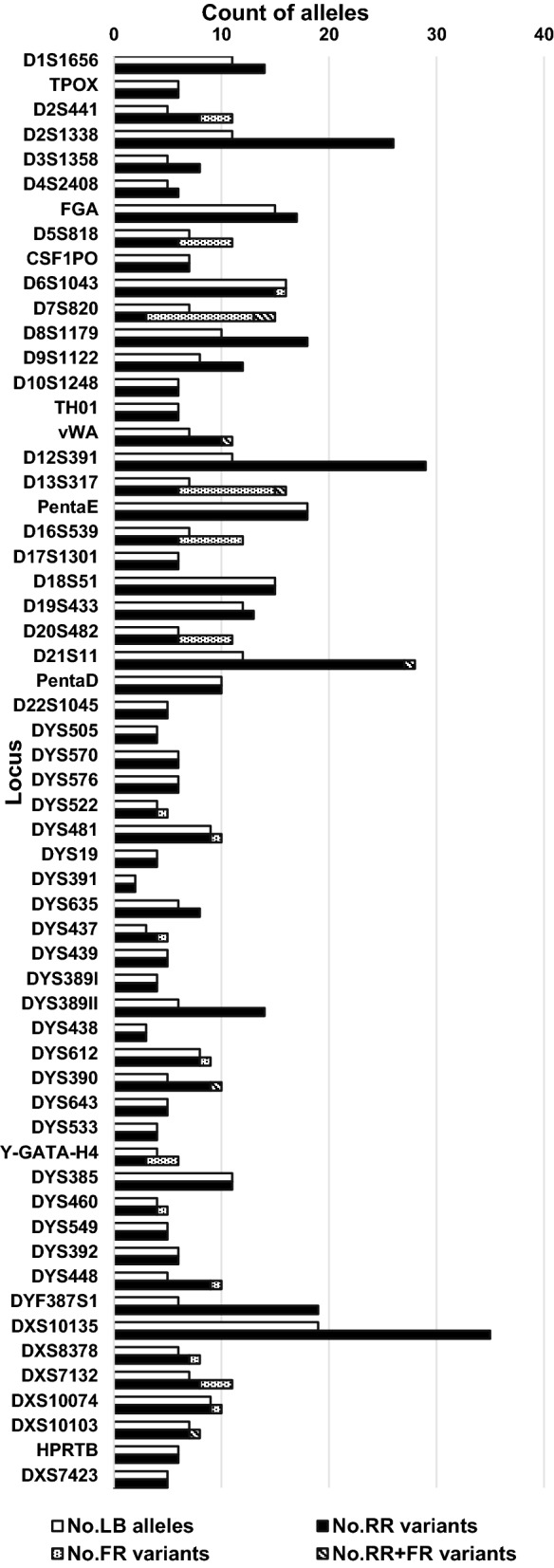
Comparison of length-based and sequence-based counting of alleles for 58 STRs. Differential shading in the columns indicates the number of alleles based on length (white), the number of alleles increased based on the sequence in the repeat region only (black), the sequence in the flanking region only (dots), and the sequence in both repeat region and flanking region (stripe).

Thirty-nine novel alleles were detected at 20 loci in the Tibetan population compared with the records in STR Sequencing Project (STRseq)^44^ (Table 1). As shown in Supplementary Table S2 (descending order), a total of 353, 166 and 83 alleles were identified through sequence-based approaches, while 241, 125 and 59 alleles were identified by length-based approaches for A-STRs, Y-STRs and X-STRs, respectively. An increase in the allele number by sequencing was observed at 17 A-STRs, 11 Y-STRs, and 5 X-STRs, in which 10 STRs showed greater than 100% (from 100 to 216.67%) increases. In concordance with that of Wang et al.’s study of 58 Tibetans^45^, STRs with increasing allele numbers were mainly compound and complex repeat STRs. We categorized the sequence variations into three groups, i.e., repeat region variants only (RRVO), flanking region variants only (FRVO) and repeat region plus flanking region variants (RRFR) (Fig. 2). We found that RRVO accounted for the largest number of variations that contributed to the increased number of alleles (Supplementary Table S2).

**Table 1:** Novel alleles observed in 107 Tibetan samples.

Locus	LB Allele	Allele name (using nomenclature according to Parson et al. 2016)	Count
FGA	26	FGA [CE26]-Chr4-GRCh38 154,587,736–154,587,823 [GGAA]2 GGAG [AAAG]16 AGAG AAAG AGAA AAAA [GAAA]3	1
FGA	27	FGA [CE27]-Chr4-GRCh38 154,587,736–154,587,823 [GGAA]2 GGAG [AAAG]17 AGAG AAAG AGAA AAAA [GAAA]3	1
FGA	29	FGA [CE29]-Chr4-GRCh38 154,587,736–154,587,823 [GGAA]2 GGAG [AAAG]19 AGAG AAAG AGAA AAAA [GAAA]3	1
D6S1043	16	D6S1043 [CE16]-Chr6-GRCh38 91,740,225–91,740,272 [ATCT]16	1
D6S1043	19.3	D6S1043 [CE19.3]-Chr6-GRCh38 91,740,225–91,740,272 [ATCT]5 ATGT [ATCT]2 ATC [ATCT]11 91,740,273-A	1
D7S820	10.1	D7S820 [CE10.1]-Chr7-GRCh38 84,160,226–84,160,277 [TATC]10 84,160,204-A; 84,160,204.1A	1
D7S820	11	D7S820 [CE11]-Chr7-GRCh38 84,160,226–84,160,277 [TATC]9 TGTC TATC 84,160,204-A	1
D8S1179	8	D8S1179 [CE8]-Chr8-GRCh38 124,894,865–124,894,916 [TCTA]7 TCAA	1
vWA	16	vWA [CE16]-Chr12-GRCh38 5,983,977–5,984,044 [TAGA]10 [CAGA]5 TAGA	4
vWA	17	vWA [CE17]-Chr12-GRCh38 5,983,977–5,984,044 [TAGA]12 [CAGA]4 CAGA	1
D12S391	25	D12S391 [CE25]-Chr12-GRCh38 12,297,020–12,297,095 [AGAT]16 [AGAC]3 AGAA [AGAC]4 AGAT	2
D16S539	8	D16S539 [CE8]-Chr16-GRCh38 86,352,702–86,352,745 [GATA]8 86,352,692-G	3
D16S539	8	D16S539 [CE8]-Chr16-GRCh38 86,352,702–86,352,745 [GATA]8 86,352,761-C	1
D19S433	12.2	D19S433 [CE12.2]-Chr19-GRCh38 29,926,235–29,926,298 [CCTT]11 cctt CCTT tt CCTT	1
D21S11	28	D21S11 [CE28]-Chr21-GRCh38 19,181,973–19,182,099 [TCTA]5 [TCTG]5 [TCTA]3 ta [TCTA]2 tca [TCTA]2 tccata [TCTA]11	2
D21S11	29	D21S11 [CE29]-Chr21-GRCh38 19,181,973–19,182,099 [TCTA]7 [TCTG]4 [TCTA]3 ta [TCTA]3 tca [TCTA]2 tccata [TCTA]10	1
D21S11	29	D21S11 [CE29]-Chr21-GRCh38 19,181,973–19,182,099 [TCTA]6 [TCTG]5 [TCTA]3 ta [TCTA]3 tca [TCTA]2 tccata [TCTA]10 19,182,101-T	2
D21S11	32.2	D21S11 [CE32.2]-Chr21-GRCh38 19,181,973–19,182,099 [TCTA]5 [TCTG]7 [TCTA]3 ta [TCTA]3 tca [TCTA]2 tccata [TCTA]11 TA TCTA	1
D21S11	33.2	D21S11 [CE33.2]-Chr21-GRCh38 19,181,973–19,182,099 [TCTA]5 [TCTG]6 [TCTA]3 ta [TCTA]4 tca [TCTA]2 tccata [TCTA]12 TA TCTA	2
DYS481	29	DYS481 [CE29]-ChrY-GRCh38 8,558,337–8,558,402 [CTT]29 8,558,336-T	1
DYS612	36	DYS612 [CE36]-ChrY-GRCh38 13,640,728–13,640,835 [CCT]5 CTT [TCT]4 CCT [TCT]25 13,640,861-C	1
DYS390	23	DYS390 [CE23]-ChrY-GRCh38 15,163,067–15,163,162 [TAGA]4 CAGA [TAGA]9 [CAGA]9 15,163,163-C	1
DYS390	26	DYS390 [CE26]-ChrY-GRCh38 15,163,067–15,163,162 [TAGA]4 CAGA [TAGA]12 [CAGA]9	2
DYS390	27	DYS390 [CE27]-ChrY-GRCh38 15,163,067–15,163,162 [TAGA]4 CAGA [TAGA]13 [CAGA]9	1
DYS390	27	DYS390 [CE27]-ChrY-GRCh38 15,163,067–15,163,162 [TAGA]4 CAGA [TAGA]12 [CAGA]10	3
Y-GATA-H4	10	Y-GATA-H4 [CE10]-ChrY-GRCh38 16,631,673–16,631,720 [TCTA]10 16,631,756-G	7
Y-GATA-H4	11	Y-GATA-H4 [CE11]-ChrY-GRCh38 16,631,673–16,631,720 [TCTA]11 16,631,756-G	15
Y-GATA-H4	12	Y-GATA-H4 [CE12]-ChrY-GRCh38 16,631,673–16,631,720 [TCTA]12 16,631,756-G	1
DYS460	9	DYS460 [CE9]-ChrY-GRCh38 18,888,956–18,888,995 [CTAT]9 18,888,914-T; 18,888,949-T	1
DYS448	17	DYS448 [CE17]-ChrY-GRCh38 22,218,923–22,219,078 [AGAGAT]10 N42 [AGAGAT]7	4
DYS448	17	DYS448 [CE17]-ChrY-GRCh38 22,218,923–22,219,078 [AGAGAT]9 N42 [AGAGAT]8	1
DYS448	18	DYS448 [CE18]-ChrY-GRCh38 22,218,923–22,219,078 [AGAGAT]11 N36 [AGAGAT]8 22,218,995–22,219,000 DEL	1
DYF387S1	39	DYF387S1 [CE39]-ChrY-GRCh38 25,884,581–25,884,724 [CTTT]18 [CTTC]8 [CTTT]2 CTTC [CTTT]2 [CTTC]4 CTAC [CTTT]3	2
DXS8378	10	DXS8378 [CE10]-ChrX-GRCh38 9,402,262–9,402,301 [ATAG]10 9,402,257-G	1
DXS8378	10	DXS8378 [CE10]-ChrX-GRCh38 9,402,262–9,402,301 [ATAG]5 ACAG [ATAG]4	1
DXS7132	10	DXS7132 [CE10]-ChrX-GRCh38 65,435,647–65,435,702 [TAGA]10	1
DXS10074	15.3	DXS10074 [CE15.3]-ChrX-GRCh38 67,757,345–67,757,400 [AAGA]10 AAA [AAGA]2 AAGG [AAGA]2	4
DXS10074	16.3	DXS10074 [CE16.3]-ChrX-GRCh38 67,757,345–67,757,400 [AAGA]11 AAA [AAGA]2 AAGG [AAGA]2	2
DXS10103	21	DXS10103 [CE21]-ChrX-GRCh38 134,284,959–134,285,038 [TAGA]2 ctga CAGA [TAGA]13 [CAGA]4 TAGA	4

Twenty-five SNPs and two InDels were detected in the flanking region of 21 STRs (Table 2), in which four SNPs and one deletion from four STRs (DYS437, Y-GATA-H4, DYS460, and DYS448) had not been previously reported in the 1,000 Genomes dataset (1,000 Genomes, https://genome.ucsc.edu/). The highest increased allele number due to flanking region variations were observed at D7S820, whose alleles with SNPs accounted for 80% of the total kinds of SB alleles. In addition, in the 214 alleles detected in D7S820, 94.39% were observed with flanking region SNPs. D13S317 presented the second highest increase with an FRV ratio of 62.50% and 55.14% of the alleles being observed with flanking SNPs.

**Table 2:** SNPs and InDels observed in STR flanking regions.

Position*	Locus	Chromosome	STR locus	Upstream/downstream	Allele	Frequency
68,011,922	rs74640515	2	D2S441	Upstream	G>A	0.0888
123,775,552	rs73801920	5	D5S818	Upstream	C>A	0.1636
91,740,273	rs529713981	6	D6S1043	Downstream	G>A	0.0047
84,160,204	rs7789995	7	D7S820	Upstream	T>A	0.9439
84,160,286	rs16887642	7	D7S820	Downstream	G>A	0.1869
84,160,205–84,160,212	rs1463708262	7	D7S820	Upstream	Dup A (insertion)	0.0047
5,983,970	rs75219269	12	vWA	Upstream	A>G	0.1916
82,148,069	rs9546005	13	D13S317	Downstream	A>T	0.5514
82,148,073	rs202043589	13	D13S317	Downstream	A>T	0.0794
86,352,692	rs563997442	16	D16S539	Upstream	C>G	0.0140
86,352,761	rs11642858	16	D16S539	Downstream	A>C	0.3551
4,525,681	rs561985213	20	D20S482	Upstream	G>A	0.0093
4,525,680	rs77560248	20	D20S482	Upstream	C>T	0.1308
19,182,101	rs1051967683	21	D21S11	Downstream	C>T	0.0093
7,547,499	rs371507752	Y	DYS522	Upstream	C>T	0.0189
8,558,336	rs370750300	Y	DYS481	Upstream	G>T	0.0189
12,346,421	NULL**	Y	DYS437	Downstream	G>A	0.1698
13,640,861	rs555095027	Y	DYS612	Downstream	T>C	0.0189
15,163,163	rs758940870	Y	DYS390	Downstream	T>C	0.0189
16,631,756	NULL	Y	Y-GATA-H4	Downstream	A>G	0.4340
18,888,914	NULL	Y	DYS460	Upstream	A>T	0.0189
18,888,949	NULL	Y	DYS460	Upstream	C>T	0.0189
22,218,995–22,219,000	NULL	Y	DYS448	Repeat region (Not counted)	Del ATAGAG	0.0189
9,402,257	rs867174547	X	DXS8378	Upstream	A>G	0.0062
65,435,703	rs778986795	X	DXS7132	Downstream	C>T	0.0435
67,757,322	rs56195635	X	DXS10074	Upstream	C>G	0.0062
134,284,967	rs754666041	X	DXS10103	Repeat region (Not counted)	C>T	0.0062

*: GRCh 38.

**: No record in dbSNP and 1,000 Genomes.

### Allele frequencies and population genetic parameters of STRs

Both LB and SB allele frequencies and other parameters for each STR locus are listed in Supplementary Tables S3–S8. The frequencies of X-STR alleles in females and males were analysed together since no significant differentiation was observed (p > 0.05). For autosomal loci and X-chromosome loci (females only), both LB and SB allele data met Hardy–Weinberg equilibrium (HWE) expectations after Bonferroni correction (A-STRs: α′ = 0.05/26, X-STRs: α′ = 0.05/7) (Supplementary Table S4). No significant linkage disequilibrium (LD) was detected in 26 A-STRs for both LB data and SB data after Bonferroni correction (α′ = 0.05/325) (Supplementary Table S5). For X-STRs and A-STRs, 20 and 23 pairs showed significant LD with SB and LB data for female samples (p < 0.05), respectively, and no LD was detected after Bonferroni correction (α′ = 0.05/528) (Supplementary Table S5).

Loci with increased number of alleles in SB data compared with LB data also showed gains in observed heterozygosities (H_obs_) (Supplementary Table S6), which is a sign of an increase of genetic diversity at these loci. The top three loci that had the largest percentage of increase in H_obs_ were D3S1358 (17.15%), D2S441 (13.90%) and D5S818 (10.39%) successively.

As shown in Supplementary Table S7, a distinct increase in total discrimination power (TDP) and a decrease in cumulative random match probability (CMP) could be observed due to the increasing diversity of SB alleles. The CMP of SB approaches for 26 A-STRs were four orders of magnitude lower than those of LB approaches. The cumulative probability of exclusion of duos (CPE_duo_) and trios (CPE_trio_) using SB data were higher than those using LB data (Supplementary Table S8). The high strength of evidence indicated the reliability of the 26 A-STRs in both personal identification and parentage testing of duos and trios (Table 3).

**Table 3:** Combined forensic parameters of datasets used in this study.

	26 A-STRs (length based)	26 A-STRs (sequence based)	94 iiSNPs (target SNPs)	94 iiSNPs (full sequences)	26 A-STRs (length based) + 94 iiSNPs (target SNPs)	26 A-STRs (sequence based) + 94 iiSNPs (full sequences)
CMP	1.943E−30	3.218E−34	6.319E−35	3.351E−37	1.223E−64	1.074E−70
TDP	1−(1.943E−30)	1−(3.218E−34)	1−(6.319E−35)	1−(3.351E−37)	1−(1.223E−64)	1−(1.074E−70)
CPE_duo_	0.9999996	0.99999998	0.9999	0.99995	0.99999999995	0.9999999999992
CPE_trio_	0.99999999996	0.9999999999995	0.99999995	0.999999993	1−(2.031E−18)	1−(3.802E−21)

For Y-STRs loci, the value of genetic diversity (GD) ranged from 0.2046 (DYS391) to 0.8672 (DYS481) and 0.2046 (DYS391) to 0.9296 (DYF387S1) when using LB data and SB data, respectively. The increased percentage of GD values of SB data compared with that of LB data ranged from 0 to 77.08% (DYS437). A total of 50 haplotypes were observed in both LB and SB data, with a haplotype diversity (HD) of 0.9971 and 48 haplotypes were unique (0.96) (Supplementary Table S9).

### Identity-informative SNPs

Forty-seven alleles with two or more SNPs within the full sequences combining the target SNPs and the flanking regions were observed at 31 iiSNP loci. Among the 47 alleles, one allele had four SNPs, six alleles had three SNPs, and the other 40 alleles had two SNPs (Supplementary Table S10). A total of 226 different sequence strings, or to say, alleles were observed based the analysis of full sequences at the 94 iiSNPs, and altogether 38 more alleles were identified compared with the analysis only based on target SNPs. Details of allele frequencies for each type of data were shown (Supplementary Table S11). The HWE test indicated that the 94 iiSNP loci (either based on target SNPs or full sequences) were in Hardy–Weinberg equilibrium after Bonferroni correction (α' = 0.05/94) (Supplementary Table S12). Five pairs and three pairs of loci showed LD after Bonferroni correction (α' = 0.05/4,371) when data based on target SNPs and data based on full sequences were considered, respectively (Supplementary Table S13). These loci were all positioned on different chromosomes, thus, we considered the 94 iiSNPs as independent for the following statistics.

The related forensic parameters for the SNPs were shown in Supplementary Table S14. The combined parameters for the data based on target SNPs and full sequences can be referred in Table 3. The strength of evidence was higher when adjacent flanking region variations of target SNPs in full sequences were taken into consideration. Observed heterozygosities showed improvements in data of full sequences compared with data of target SNPs (Supplementary Table S15). The effective number of alleles (Ae), an important and effective index for evaluation of the selection of microhaplotypes for mixture detection^46^, also showed some level of increases in data based on full sequences when compared with the Ae of corresponding data of target SNPs only (Supplementary Table S15). For data of full sequences, ten loci had an Ae value > 2.00, of which rs1109037 and rs10776839 had values > 3.00, which was a necessary criterion for microhaplotypes being applied to mixture detection^46^. For the target SNP data, in contrast, only five loci showed an Ae > 2.00.

When we combined the length-based data of 26 A-STRs with data from the target SNPs of 94 iiSNPs, eight of 7,140 pairwise comparisons still showed LD (p < 0.00001) after Bonferroni correction (α' = 0.05/7,140) (Supplementary Table S16). Regarding the combination of sequence-based data from 26 A-STRs and data from full sequences of 94 iiSNPs, the same number of pairwise comparisons showed LD (p < 0.00001) after Bonferroni correction (α' = 0.05/7,140) (Supplementary Table S16). None of these pairwise comparisons with significant LD were syntenic. Relative forensic parameters were shown in Table 3. The power for personal identity of the 94 iiSNPs was three to five magnitudes higher than the 26 A-STRs, while in the case of the ability of parentage testing of duos and trios, the 26 A-STRs were higher than the 94 iiSNPs. Moreover, when combining A-STR and SNP markers, the power of the system efficiency was much higher (about 30 to 35 magnitudes lower for CMP and four to ten times higher for CPE) than detection using one category of markers only.

## Discussion

The Tibetan population described in this study exhibited many sequence variations in repeat regions and flanking regions based on MPS data. A total of 33 STRs showed a higher diversity of alleles when considering sequence variations rather than considering length-based alleles only, while 25 loci showed no increase in allele number by the SB method. Thirty-nine novel alleles were detected, although only 107 samples were studied. Twenty-five SNPs and two InDels were detected in the flanking regions of 21 STRs. InDels existing in the flanking regions of sequences may influence the length call definitions of alleles. Variants with a substantially differences in frequency distributions between different populations is an indicator of the ancestry-informative value of the locus^47^. As for iiSNPs, compared with the alleles focused on target SNPs, 47 alleles with two or more SNPs within the full sequences combining both target SNPs and the flanking regions were observed at 31 iiSNP loci. Similar results of the heterozygote imbalance of D22S1045 were reported by Novroski^34^, Churchill^48^, Just^31^ and Hussing^38^, and Hussing et al. also chose not to analyse this locus further in Danes^38^. While in Novroski’s and Churchill’s reports, the number of SB alleles of D22S1045 were increased due to FR variations, but in Chinese populations (^45,49^ and our study), no sequence variation (neither repeat nor flanking region) was observed at the D22S1045 locus. Overall, the sequence variations observed herein were consisted with the observations reported in previous literature^34,37,39,45,50^.

Five pairs and three pairs of loci showed LD after Bonferroni correction when data of target SNPs and full sequences of iiSNPs were considered, respectively. Referring to previous similar studies^10,38,51,52^, it was not surprising to observe the LD phenomenon for iiSNPs pairs. These loci were considered as independent when calculating forensic parameters since these iiSNP pairs were located on different chromosomes^38,52^. We suppose it was the special population structure of the aimed population group that caused the disequilibrium. Meanwhile, considering the small sample size of this study, failure of LD testing may also result from random effect.

Aiming to correctly interpret the complex data produced by MPS platforms, a convenient and sophisticated software package for data analysis may promote the use of MPS platforms for this type of forensic genetic study. The two software packages we used here for concordance analysis have their own characteristics. As an offline software with customized web browser interface for forensic use, UAS v1.2.16173 can report the LB allele callings for STRs and genotypes of target SNPs, and can calculate RMP and TDP for specific populations. Although this version of UAS doesn’t support flanking region analysis, it has been improved and allowed the analysis in the upgraded version (UAS v1.3 or later). STRait Razor v2s can analyse more than 300 loci (including STRs, SNPs and InDels), and focus on both repeat regions and flanking regions of the investigated loci, and can report a SB allele in concordance with the minimal nomenclature requirements recommended by ISFG. Moreover, a much more informative form of SNPs (alleles based on full sequences) can be obtained using an MPS platform rather than alleles based on target SNPs only, which may facilitate mixture deconvolution in the future.

**Figure 3:**
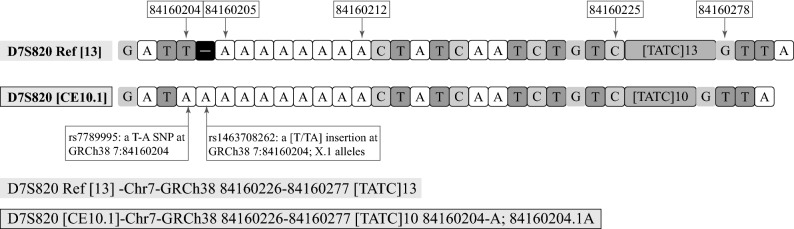
Instances of InDel in D7S820.

In order to adapt to the backward compatibility of the CE typing system, the nomenclature recommended by ISFG^21^ contains repeat number information based on allele length. Similar to the principles of the CE method, the ‘CE callings’ for SB alleles were determined by comparing the length of the fragment with the same structure length relative to a reference sequence. It is important to note that the CE callings may not represent the actual numbers of repeat units of an allele, especially in alleles with flanking region variations. The annotation of the flanking variants in the nomenclature can indicate the true status of a sequence, which is important for researchers to quickly determine the diversity of a given sequence. An InDel that exists in a flanking region may not be identified but can influence the length of a fragment. In this study, an InDel at D7S820 could explain the influence of InDels on nomenclature (Fig. 3). Through the sequence structure, a [T/TA] insertion (provisionally rs1463708262 (GRCh38 7:84,160,205–84,160,212)) was identified, which resulted in the allele length recognized by STRait Razor v2s as 1 nt longer than the length of allele 10. Thus, the CE number was termed 10.1. Meanwhile, a T-A transversion (rs7789995) was identified close to the insertion (GRCh38 7:84,160,204), which had allele frequencies of 99%, 91%, 94%, 87%, 92% in African, American, East Asian, European, South Asian, respectively (1,000 Genomes, https://genome.ucsc.edu/). Hence, the SB allele name of the string sequence was “D7S820 [CE10.1]-Chr7-GRCh38 84,160,226–84,160,277 [TATC]10 84,160,204-A; 84,160,204.1A”, regardless of if the actual number of repeat units was 10. Moreover, the exact position of the A insertion in the above example was ambiguous. The insertion may exist at any position from 84,160,204 to 84,160,212. Consistent with this observation, the possible InDels have not been defined in the reference template yet^53^. Similar allele callings were observed in 11 loci (D7S820, D13S317, PentaD, DYS385, DYS460, DYS448, DXS10135, DXS10074, HPRTB and DXS7423). This type of inconsistency between alleles and sequences was reported by Novroski et al.^34^

Moreover, discrepancies in SB allele nomenclature could be observed when using different coordinates of sequence guides and analysis tools. In previous studies of SB alleles, some researchers followed the nomenclature recommended by ISFG^21^, while others used custom-defined nomenclature^36,37^. Discordant nomenclatures can lead to inconsistent allele calling between laboratories, further confusing precise allele and InDel calling between populations. An optimised consolidation of allele nomenclature and reference genome coordinates for SB alleles should be addressed for a convenient method for data communication between laboratories is urgently needed.

Lastly, a high system efficiency of the selected 58 STRs and 94 iiSNPs was demonstrated in this Tibetan population. MPS methods make the combined detection of STRs and SNPs more convenient, thereby improving the system efficiency dramatically. Cases involving personal identification and parentage testing of duos and trios can thus be solved with reliable results. The combination of detection of STRs and SNPs may help to solve problems in complex kinship analysis more efficiently. A lofty goal of this field would be reducing the number of markers (or removing loci with low diversity) when conducting duo and trio testings at an acceptable performance level. Furthermore, exploring customized marker subsets for different identification purposes is also an area of future interest.

## Conclusions

This study investigated sequence polymorphisms of 58 STRs and 94 iiSNPs in a Tibetan population using massively parallel sequencing, and provided an accurate sequence-based allele frequencies dataset for these loci. Distinct sequence variations were observed in both repeat and flanking regions of these loci, which indicated that the ForenSeq DNA Signature system is highly polymorphic and informative in the studied population. Our study also demonstrated a potential capability for this system to be applied in kinship testing and personal identification cases.

## Materials and methods

### Sample collection

Peripheral blood samples were collected using FTA cards from 107 unrelated individuals (53 males and 54 females) from Wei Tibetan population in the Tibetan Autonomous Region of western China.

### Library preparation and sequencing

DNA libraries were constructed using the ForenSeq DNA Signature Prep Kit according to the manufacturer’s recommendations^54^. Briefly, 1.2 mm diameters of FTA cards were punched directly as an input template without DNA extraction. Target amplification and tagging were performed under advised thermal cycling parameters. Index 1 (i7) and index 2 (i5) adapters were added for target enrichment purposes. Then the libraries were purified using Sample Purification Beads (SPB) and normalized using Library Normalization Beads 1 (LNB1). Finally, 5 μL of the normalized library from each sample was pooled into a single microcentrifuge tube. Seven microliters of pooled libraries were added into 591 μL Hybridization Buffer (HT1) and mixed with 2 μL of diluted Human Sequencing Control (HSC) mixture. Sequencing was performed on a MiSeq FGx instrument (Illumina, San Diego, CA) using the MiSeq FGx Reagent Kit (Illumina, San Diego CA) following the manufacturer’s instructions. Two runs were performed to cover all samples in this study.

### Data analysis, allele nomenclature, and sequence variant identification

The raw sequencing data of STRs was first analysed using ForenSeq UAS (v1.2.16173) with default analytical and interpretation thresholds (AT = 1.5%, IT = 4.5% in general, respectively) for allele calling^17^. The intralocus balance threshold was measured as the intensity (number of reads) of the minimum intensity typed allele divided by the intensity of the maximum intensity typed allele and was set as 0.60 (default setting) for all loci except for D22S1045 (intralocus balance threshold = 0.10), which was suggested to be analysed with caution by the manufacture due to the extreme heterozygote imbalance. Alleles were reported using length calling and sequence calling, which contain the repeat region of the locus.

The actual ACR was determined for heterozygote loci by dividing the lower number of reads by the higher number of reads. Then, the FASTQ files were re-analysed by STRait Razor v2s^12^ software with the default analytical threshold (AT = 2 reads) and heterozygote threshold (HT = 0.40, the same meaning with intralocus balance threshold). In-house workbooks (written by VBA using Microsoft Excel) were developed to modify the format of the nomenclature produced by STRait Razor v2s so as to completely conform to the requirements recommended by ISFG^21^ and the revised Forensic STR Sequence Guide_v3^53^. Manual corrections were also performed to verify the nomenclature of SB alleles identified as ‘novel’ by STRait Razor v2s. The 94 iiSNPs were genotyped using ForenSeq UAS with default settings (AT = 1.5%, IT = 4.5%), from which we obtained the alleles and genotypes according to the target SNPs. Comprehensive nomenclature following Parson et al.^21^ were obtained using STRait Razor v2s^12^, from which we obtained the alleles and genotypes considering the full sequences of SNPs.

**Figure 4:**
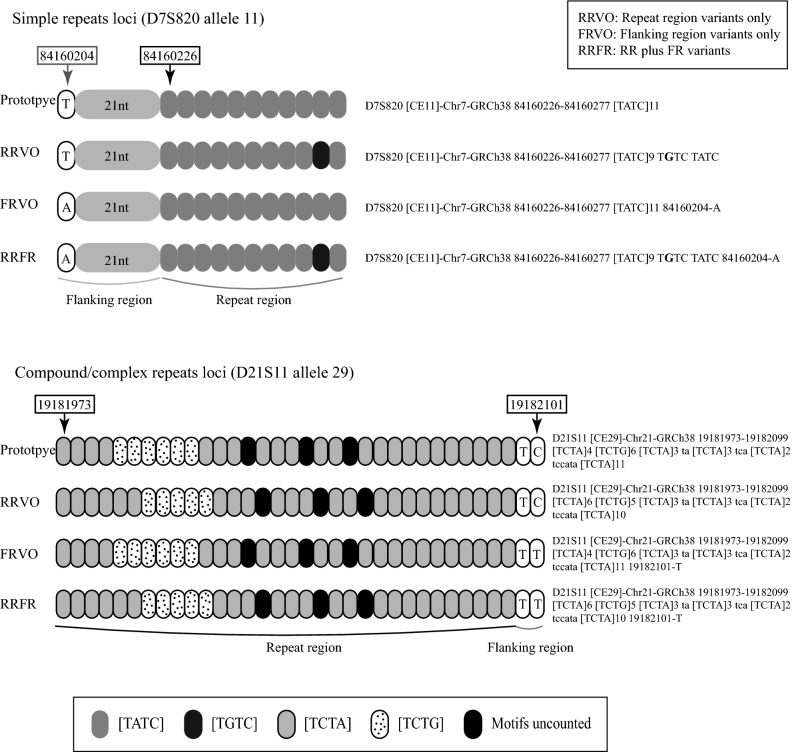
Instances of reference alleles and three categories of variants. RRVO represents the internal sequence variations present in repeat region which is different from the reference allele. FRVO represents the sequence variations with flanking region variations only, while the repeat region is the same as the reference allele. RRFR stands for the sequence with both repeat motif variations and flanking region variations.

The allele sequence variants of STRs were classified into three categories: repeat region variants only (RRVO), flanking region variants only (FRVO), and repeat region plus flanking region variants (RRFR) (Fig. 4). Reference alleles were defined using the STRBase database (https://strbase.nist.gov/str). Novel alleles were newly discovered if they had not been previously reported in the STR Sequencing Project (STRseq, https://www.ncbi.nlm.nih.gov/bioproject/PRJNA380127, accessed: 18 October 2018)^44^. The SNPs and InDels in flanking regions were compared to the UCSC Genome Browser (1,000 Genomes, https://genome.ucsc.edu/) and were also verified in the NCBI database (dbSNP, 152 build, https://www.ncbi.nlm.nih.gov/snp/).

The genotyping data of A-STRs and corresponding string sequences were submitted to STRidER (https://strider.online/) for quality control^22^.

### Capillary electrophoresis and concordance analysis

All samples were genotyped using the Goldeneye DNA ID System 25A amplification system. The system contained the 20 expanded Combined DNA Index System (CODIS) core loci plus Penta E, Penta D, D6S1043, a Y indel and Amelogenin for sex identification. DNA amplification was performed according to the manufacturer’s instructions. The PCR products were detected using CE on an ABI 3500xL Genetic Analyzer (Applied Biosystems, USA). The results were analysed with GeneMapper ID-X Analysis Software (Applied Biosystems, USA). Concordance analysis was performed between the LB alleles produced by Miseq and the corresponding CE results. The comparison of genotyping results from UAS and STRait Razor v2s was also performed.

### Allele frequencies and forensic parameters

A counting method was utilized to obtain the LB and SB allele frequencies. For A-STRs, a HWE test and pairwise LD analysis was performed using Arlequin 3.5.2.2^55^. The expected and observed heterozygosity (H_exp_, H_obs_), polymorphism information content (PIC), discrimination power (DP), random match probability (RMP), and power of exclusion (PE) for both duos and trios of the 27 A-STRs were calculated with Cervus 3.0.7^56^. For X-STR loci, the differentiation of the X-STR allele frequency distribution of females and males was performed using Arlequin 3.5.2.2^55^. HWE test and LD analysis were performed for X-STRs of females. The mean exclusion chance for father/daughter duos (MEC_duo_) and father/mother/daughter trios (MEC_trio_) were calculated on the ChrX-STR.org.2.0 website (https://chrx-str.org/)^57^. Finally, an LD test for the 27 A-STRs combined the 7 X-STRs of females was performed using Arlequin 3.5.2.2^55^. Relevant Y-STR parameters, which included genetic diversity (GD), haplotype diversity (HD), allele frequencies and haplotype frequencies, were calculated using an in-house workbook (written by VBA using Microsoft Excel). The formulas were as follows:

1 $${\text{GD}} = [{\text{n}}*({1} - \sum {\text{p}}_{i}^{2} )]/\left( {{\text{n}} - {1}} \right),$$

2 $${\text{HD}} = [{\text{N}}*({1} - \sum {\text{p}}_{j}^{{2}} )]/\left( {{\text{N}} - {1}} \right),$$

where n represents the number of alleles, p_*i*_ represents the allele frequency, N represents the number of haplotypes, and p_*j*_ represents the haplotype frequency.

HWE and LD for both target SNPs and full sequences of iiSNPs were performed using Arlequin 3.5.2.2^55^. Cervus 3.0.7^56^ was used to calculate allele/full sequence frequencies, H_exp_, H_obs_, PIC, DP, RMP, PE_duo_ and PE_trio_. The effective number of alleles (A_e_) was defined as the reciprocal of the homozygosity:

3 $${\text{A}}_{{\text{e}}} = {1}/\sum {\text{p}}_{i}^{2} ,$$

where p_i_ represents the frequency of the *i*th allele according to Kidd^46^. We also evaluated the performance of combination of LB A-STRs and target iiSNPs, SB A-STRs and full sequence of iiSNPs with the same methods.

### Ethics statement

All of the experimental process in this study were strictly followed the ethical research principles, and all methods following were performed in accordance with the relevant guidelines and regulations. All samples were anonymously collected after informed consent was obtained. This study was approved by the Ethics Committee of Sun Yat-sen University with the approval number of No. 11[2012].

## Supplementary information

Supplementary Tables

## Data Availability

The datasets generated and analysed during the current study are available from the corresponding author on reasonable request.
